# Accurate magnetic field imaging using nanodiamond quantum sensors enhanced by machine learning

**DOI:** 10.1038/s41598-022-18115-w

**Published:** 2022-09-01

**Authors:** Moeta Tsukamoto, Shuji Ito, Kensuke Ogawa, Yuto Ashida, Kento Sasaki, Kensuke Kobayashi

**Affiliations:** 1 Department of Physics, The University of Tokyo, Bunkyo-ku, Tokyo, 113-0033 Japan; 2 Institute for Physics of Intelligence, The University of Tokyo, Bunkyo-ku, Tokyo, 113-0033 Japan; 3 Trans-scale Quantum Science Institute, The University of Tokyo, Bunkyo-ku, Tokyo, 113-0033 Japan

**Keywords:** Quantum metrology, Nanoparticles

## Abstract

Nanodiamonds can be excellent quantum sensors for local magnetic field measurements. We demonstrate magnetic field imaging with high accuracy of 1.8 $\upmu$T combining nanodiamond ensemble (NDE) and machine learning without any physical models. We discover the dependence of the NDE signal on the field direction, suggesting the application of NDE for vector magnetometry and the improvement of the existing model. Our method enhances the NDE performance sufficiently to visualize nano-magnetism and mesoscopic current and expands the applicability of NDE in arbitrarily shaped materials, including living organisms. This accomplishment bridges machine learning to quantum sensing for accurate measurements.

## Introduction

**Figure 1:**
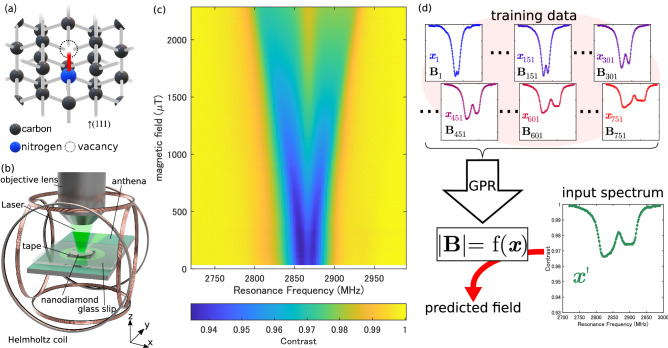
Implementation of nanodiamond quantum sensors enhanced by machine learning. (**a**) Schematic of a nitrogen-vacancy (NV) center in diamond. (**b**) Experimental setup. The optical axis is the z-axis, and the NDE is spread on the surface in the xy-plane. (**c**) Experimentally obtained ODMR spectra of NDE as functions of the microwave frequency and the magnetic field. The true magnetic field is measured using a tesla meter. (**d**) Schematic of our machine learning method. The ODMR spectrum and the true magnetic field of (**c**) are used as the input vector $\varvec{x}_i$ and output scalar $y_i$ for training, respectively. Using GPR, a function is obtained from the training data to predict the magnetic field strength $|\mathrm {\varvec{B}}| = f(\varvec{x}')$ from an unknown spectrum $\varvec{x}'$.

Local magnetometry is a crucial technology for characterizing nano- and micro-materials and has been implemented using various scanning techniques^1,2^ or diamond quantum sensors^3–7^. The nitrogen-vacancy (NV) center in diamond (Fig. 1a) is a point defect where a nitrogen atom replaces a carbon atom in the lattice accompanied by a neighboring vacancy. We can detect the electron spin resonance of the NV center by measuring its photoluminescence intensity while irradiating the laser and microwave, called optically detected magnetic resonance (ODMR)^8^. As the spin level of the NV center splits against the magnetic field in the direction of the NV symmetry axis (111) due to the Zeeman effect^9^, the determination of the ODMR frequency serves as quantum sensing of the field^3^. To obtain a nanoscale spatial resolution, we must attach the NV centers close to the sample within a few 10 nm^10^. For this purpose, scanning techniques using diamond probes^4,11^ or attachments of micro-fabricated diamond pieces^12,13^ are used.

The nanodiamonds (NDs) could be an alternative to realize a similar adjacency^14^. ND is a diamond crystal with a 5–200 nm diameter harboring NV centers^15^. Since it enables us to adhere NV centers to any materials with arbitrary shapes by simply dropping NDs dispersed in a liquid, it has been applied to measurements of electronic devices^16^.

Figure 1b shows our setup^17^ to precisely measure the magnetic field dependence of the ODMR spectrum of nanodiamond ensemble (NDE). We use a three-axis Helmholtz coil to adjust the magnetic field with a precision of ± 0.12 $\upmu$T, determined with a tesla meter (Lake Shore Cryotronics F71). Hereafter, we refer to this field value as the true magnetic field. The z-axis magnetic field dependence of the ODMR spectrum of NDE on a cover glass placed in the xy-plane is shown in the image plot of Fig. 1c. This result is obtained from a total of one million NV centers contained in about 30,000 NDs (see “Method and material”). The spectrum is measured at 751 points from 6 to 2286 $\upmu$T with a field applied in the optical (z-axis) direction. The spectral shape becomes broadened with two dips as the field increases due to Zeeman splitting, and the ODMR contrast degrades. This behavior reflects that the crystal axes of each ND are random, resulting in different resonance frequencies of NV centers.

It is significant how accurately the above method can deduce the magnetic field. While a physical model for such a randomly oriented NDE is available^14^, the complexity of actual experimental conditions still limits the accuracy. For example, we fit the spectrum obtained at the true magnetic field of 547.1 ± 0.12 $\upmu$T with the model (“Method and material”)^14^. The estimated field of 582.2 ± 4.4 $\upmu$T is close to the true field but is statistically inaccurate. This observation exemplifies the problem of hindering accurate local magnetometry using NDE. While NV centers are known to show very high sensitivity to the magnetic field—how sensitive the signal changes depending on the field modulation, the accuracy achieved by them, that is, trueness of the measured value, has been less emphasized.

Here, we demonstrate the accuracy improvement by Gaussian process regression (GPR), a model-free machine learning method^18,19^. We have successfully proven that the GPR can estimate the magnetic field more accurately than the physical model. We also reveal the dependence of the NDE signal on the field direction, suggesting the application for vector magnetometry and the improvement of the existing model. To expand the method to field imaging, we demonstrate the measurement of the Oersted field from the current flowing in a wire. Finally, we achieve the accuracy of 1.8 $\upmu$T by taking statistics for each pixel.

## Results and discussions

We use GPR, a flexible and nonparametric machine-learning protocol commonly applied to function estimation^18–20^, to deduce the magnetic field from the randomly oriented NDE. The ODMR spectrum and the true magnetic field in Fig. 1c are used for training as the input variable $\varvec{x}_i$ and output variable $y_i$, respectively, as shown in Fig. 1d. The function $f(\varvec{x})$ is obtained to estimate the magnetic field from an arbitrary spectrum $\varvec{x}$. It is characterized by the kernel function $k(\varvec{x},\varvec{x}')$, which represents the degree of agreement between two input variables $\varvec{x},\varvec{x}'$. We adopt the squared exponential kernel, the most widely used kernel function given by

1 $$\begin{aligned} k(\varvec{x},\varvec{x}') = \exp (-\theta ||\varvec{x}-\varvec{x}'||^2). \end{aligned}$$

The only two hyperparameters are the variable $\theta$ and the noise of the acquired data $\beta ^{-1}$ (“Method and material”). For a robust analysis, we normalize the contrast of the ODMR spectrum and use the data after taking the first derivative for frequency as the input variable.

**Figure 2:**
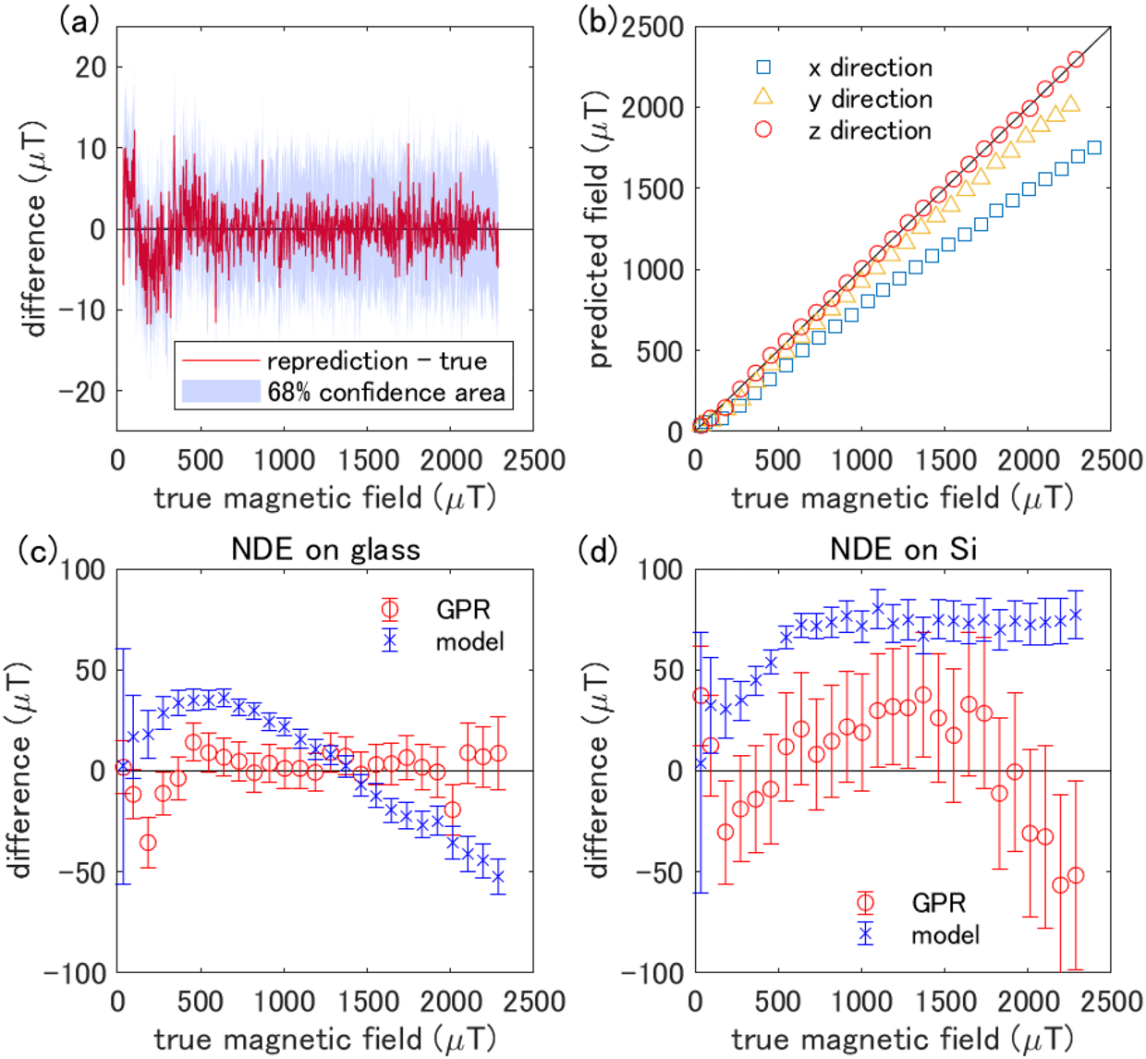
Performance evaluation of GPR and comparison with the physical model. (**a**) Benchmark of reprediction by GPR. The horizontal axis is the true magnetic field, and the vertical axis is the difference between the repredicted field and the true field. The light purple region is the standard deviation interval. (**b**) Dependence of the predicted magnetic field on the magnetic field directions. The black line is the ideal value where the predicted and true magnetic fields perfectly agree for the z-direction. (**c**,**d**) The detail of the prediction accuracy for the NDE (**c**) on the cover glass and (**d**) on the silicon wafer. We use only the training data when the field is applied to the z-direction. The difference between GPR predicted and true field is shown by a red circle. The corresponding result using the fitting based on the physical model^14^ is shown by a blue cross. The error bars depict a 68% confidential interval. Note that we have corrected the data for NDE on silicon regarding the heating of the material (see Fig.  S3 for detail).

The red curve in Fig. 2a shows the prediction of the magnetic field using the training data itself as the input variable $\varvec{x}'$ for checking the regression process. The vertical axis is the difference between the repredicted magnetic field and the true one. The light purple area is the standard deviation of the reprediction, which indicates the uncertainty of the GPR prediction of the magnetic field from the ODMR spectrum. The repredicted field agrees with the true field within a standard deviation range of approximately ± 10 $\upmu$T.

To confirm that GPR can work for quantum sensing, we deduce the estimation accuracy for a test data set not used for the training. The procedure corresponds to the evaluation of generalization performance in machine learning. Figure 2b shows the magnetic field predicted by the GPR from the test data reacquired by the NDE on the cover glass as red circles (“z-direction”), indicating that the predicted field is perfectly consistent with the true one regarding the z-direction. Figure 2c shows the difference between predicted and true values as red circles more quantitatively. The predicted values encompass the true values within their statistical uncertainty for a wide magnetic field range. Taking the case mentioned above as an example, when the true value is 547.1 ± 0.12 $\upmu$T, machine learning yields 556.1 ± 9.5 $\upmu$T. On the other hand, the fields estimated from the model fitting (blue cross) deviate from the true ones statistically and systematically, indicating a limit of the model’s accuracy due to several factors that cannot be readily determined experimentally. These results demonstrate the robustness and superior accuracy of the present model-free method compared to the conventional one.

We then verify whether GPR works for NDE on different materials. As the training data, we use the same data of the NDE on the cover glass as before. Figure 2d shows the corresponding result obtained from the NDE on a silicon wafer. Again, the GPR yields a statistically more reliable estimation than the model. Thus, we confirm that GPR works well even when it is impossible to prepare training data, such as NDE spread over magnetic materials. We note that the magnetic field derivation is more subtle in this silicon case than in the above glass case because the frequency dependence of the microwave antenna, which is difficult to be determined experimentally, is more delicate (Supplementary Information).

So far, we have discussed only the case of applying a magnetic field in the z-direction. Despite the isotropic distribution of NDs over the surface in the xy plane, the ODMR spectrum can be anisotropic concerning the field direction for several reasons, such as the numerical aperture of the objective lens (Supplementary Information). Figure 2b depicts the predicted magnetic field values when applying the magnetic field individually in the x-, y-, and z-directions. The data with the magnetic field applied in the z-direction is used as the training data for all predictions. The results systematically deviate from the true magnetic field when applying the field in the x- and y-directions, which is a different direction used for the training data. The observed anisotropy is profitable because it suggests vector field sensing with NDE. The contribution of each directional component is tunable by changing the objective lens’ numerical aperture or the direction of the excitation light’s linear polarization; In principle, the magnetic field vector could then be determined by combining these data. Also importantly, our finding directly points to improving the existing model by considering these factors.

**Figure 3:**
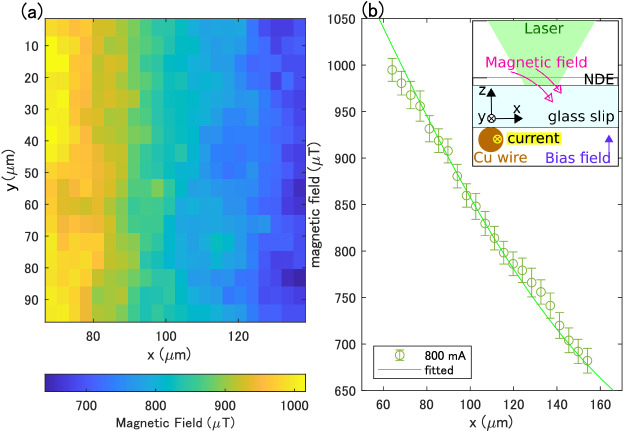
Magnetic field imaging enhanced by machine learning. (**a**) Magnetic field distribution when a current of 800 mA is applied to the copper wire, which is placed along the y-direction. The horizontal axis is the distance to the x-direction when the copper wire is placed at $x = 0\ \upmu$m. (**b**) The average magnetic field value against the y-axis direction in (**a**). The solid curve is the result of the fitting based on Ampere’s law. (Inset) Measurement configuration, where the bias field applied in the z-direction and the field generated by the current through the wire are simultaneously felt by the NDE.

Now we go back to the amplitude estimation. We can easily suppress the above direction dependence to a negligible amount by applying a bias magnetic field in the z-direction. We measure the magnetic field generated by the current flowing through a copper wire under the cover glass as a demonstration. Figure 3a shows the magnetic field distribution when 800 mA is applied to the wire with a bias field of 912.8 $\upmu$T in the z-direction. We observe that the magnetic field becomes larger as the position gets closer to the wire, consistent with Ampere’s law. For a more quantitative evaluation, the average value of the magnetic field in the y-direction is shown in Fig. 3b. The fitted results based on Ampere’s law plus the bias field are consistent with the experimental results within the error bars.

**Figure 4:**
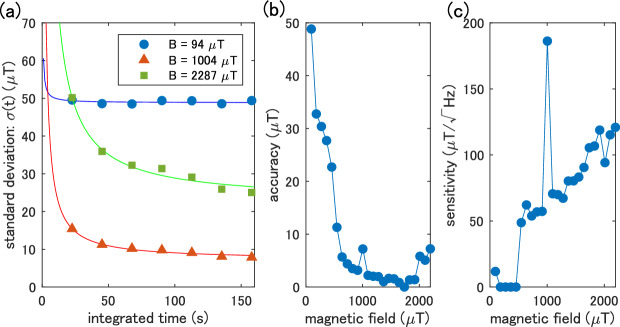
Accuracy and sensitivity of nanodiamond quantum sensors. (**a**) Standard deviation of the difference between the true and the predicted magnetic field from the test data. The horizontal axis is the measurement time for test data. (**b**,**c**) Magnetic field dependence of (**b**) accuracy and (**c**) sensitivity obtained by fitting of (**a**). An outlier near 1000 $\upmu$T is due to an accidental artifact in the analysis in terms of low signal-to-noise ratio (Section S5in Supplementary Information).

Finally, we evaluate the accuracy and sensitivity achieved in our method when the field is applied in the z-direction. We calculate the difference between the true magnetic field and the predicted value from the test data for each pixel (17 $\times$ 22 pixels, 1 pixel$~\approx 18~\upmu$m$^2$), as well as the standard deviation $\sigma$. The integration time *t* dependence is also obtained for each field. In this analysis, the integration time of both training and test data is varied similarly. As shown in Fig. 4a, as the integration time for the test data increases, the standard deviation decays. The accuracy $\zeta$ and sensitivity $\eta$ are obtained by fitting $\sigma (t) = \eta t^{-0.5} + \zeta$, considering the shot noise due to photon counting. Note that the accuracy $\zeta$ is less than the uncertainty of the function estimation $\sim 10\ \upmu$T discussed in Fig. 2a, since $\zeta = \lim _{t\rightarrow \infty } \sigma (t)$.

The magnetic field dependence of the accuracy and the sensitivity are shown in Fig. 4b,c, respectively. The better the accuracy and sensitivity are, the lower these values are. The accuracy (Fig. 4b) degrades at low field ($<500~\upmu$T) and high field ($>2000~\upmu$T). These behaviors are inevitable due to the nature of the NV centers in NDE. There is only a tiny change in the spectrum at low fields (see Fig. 1c) because splitting the ODMR spectrum due to the lattice strain obscures the magnetic field dependence^21^. The accuracy is also not good in high fields due to the decrease in the ODMR contrast because of the random distribution of NV axes in NDE. The sensitivity (Fig. 4c) also shows this tendency in high fields. The sensitivity at low fields is almost zero, which is simply an artifact due to the low resolution for integrated time (see Fig. 4a). As a result, we achieve the best accuracy of about 1.8 $\upmu$T under the field of 1000–1500 $\upmu$T with a sensitivity of $\sim 70$ ${\upmu T/\sqrt{Hz}}$.

In summary, we demonstrate the accurate local magnetometry by the machine-learning-enhanced NDE method. The high accuracy and sensitivity obtained are sufficient for the magnetic imaging of a few layers of the van der Waals materials^22–26^ and topological materials^27,28^ and for visualizing the mesoscopic current^29,30^. This method applies not only to such topics in condensed matter physics but also to arbitrary-shaped objects such as living organisms^15,31^. The present idea is further promising in applying temperature, electric field, and pressure measurements by using NDE^21,32–34^. In terms of quantum technology, machine learning has chiefly applied to quantum state estimation and protocol optimization so far. Our work opens up the future possibilities of machine learning for accurate quantum sensing.

## Method and material

### Experimental setup

A laser beam (515 nm, 150 mW) is output through a multimode fiber and a collimator into the free space. It is expanded by a lens pair before entering the objective lens (Mitsutoyo M-PLAN APO 100X, numerical aperture NA = 0.7, magnification $\times$100). The irradiated area of the excitation beam at the focal point is approximately 400 $\upmu$m in diameter. The NV center’s fluorescence is acquired by a CMOS camera (Basler acA720–520um, 12-bit resolution) after passing through an objective lens, a dichroic mirror, a 514 nm notch filter, a 650 nm long-pass filter, an 800 nm short-pass filter, and an imaging lens. The magnetic field generated in the three-axes Helmholtz coil, including the geomagnetic field, is calibrated with a tesla meter (LakeShore Cryotronics F71) to an accuracy of $\pm \, 0.12~\upmu$T.

The NDE spread on the cover glass is fixed with carbon tape on the resonator microwave antenna^35^. ODMR measurements are performed by inputting 25 dBm microwave power to the antenna.

The ODMR spectra are acquired with a frequency resolution of 141 points equally spaced between 2720 and 2990 MHz. The exposure time is set to 8 ms, and the frequency sweep is repeated 70 times (8 ms $\times$ 141 $\times$ 70 in total).

In the measurement shown in Fig. 3, a copper wire with a diameter of 50 $\upmu$m is placed along the y-axis direction. A magnetic field is generated by applying a current to the wire (Fig. 4b, inset).

### Sample preparation

NDs with a standard particle size of 50 nm (NDNV50nmHi10ml, Adámas Nanotechnologies) containing about 30 NV centers per particle are used. 5 $\upmu$g of NDs in 5 $\upmu$L of water are dispersed by ultrasonication and then dropped onto cover glass (130 $\upmu$m thick, Matsunami Glass Ind.,Ltd.) and dried with a spin coater rotating at 500 rpm for 2000 s. The thickness of NDE is obtained to be 200 nm (about 4 layers) by measuring with a profilometer (KLA-Tencor P-7). This value corresponds to about 30,000 NDs and about 1 million NV centers per image pixel (18 ${\upmu m^2}$). The fabricated NDE looks like a white film due to light scattering under ambient light. The NDE on silicon is spread with the spin coater at 400 rpm, and its thickness is obtained to be 1000 nm (about 20 layers).

### Gaussian process regression (GPR)

GPR is a flexible and nonparametric machine-learning protocols commonly applied to function estimation^18–20,36^. It is characterized by the kernel function $k(\varvec{x},\varvec{x}')$, which represents the degree of agreement between two input variables $\varvec{x},\varvec{x}'$. We adopt the most widely used kernel function,

2 $$\begin{aligned} k(\varvec{x},\varvec{x}') = \exp (-\theta ||\varvec{x}-\varvec{x}'||^2), \end{aligned}$$

which is known as the squared exponential kernel.

As training data, we prepare *n* pairs of input variable vector $\varvec{x}_i$ and output scalar variable $y_i$. When the input data is $\varvec{x'}$, the predicted output value $f(\varvec{x'})$ is given by,

3 $$\begin{aligned} f(\varvec{x}') = \varvec{k}(\varvec{x}')^{\mathrm {T}} (K + \beta ^{-1} I)^{-1} \varvec{y}, \end{aligned}$$

where *I* is an identity matrix, $\varvec{y}$ is a column vector with $y_i$ as its *i* th entry, $\varvec{k}(\varvec{x}')$ is a column vector with $k(\varvec{x}_i,\varvec{x}')$ as its *i* th entry, and *K* is an $n\times n$ matrix with $K_{i,j} = k(\varvec{x}_i,\varvec{x}_j)$ as its (*i*, *j*) th entry, $\beta ^{-1}$ is the noise intensity on the output variable *y*. In this case, standard deviation $\varvec{v}(x')$ (confidence area of Fig. 2a) is given by

4 $$\begin{aligned} \varvec{v}(x') = k(\varvec{x}',\varvec{x}') + \beta ^{-1} -\varvec{k}^{\mathrm {T}} K^{-1} \varvec{k}. \end{aligned}$$

In this study, $\varvec{x}_i$ and $y_i$ are the ODMR spectra shown in Fig. 1c and the magnetic field strength obtained with a tesla meter, respectively. The optimization of the hyperparameters is performed by the minimization of five-fold cross-validation loss using the MATLAB Statistics and Machine Learning Toolbox. According to Eq. (2), the magnetic field $f(\varvec{x}')$ is predicted by calculating the similarity between the ODMR spectrum $\varvec{x}'$ and each training data. The only two hyperparameters are the variable $\theta$ and the noise of the acquired data $\beta ^{-1}$. For a robust analysis, we normalize the contrast of the ODMR spectrum and use the data after taking the first derivative for frequency as the input variable.

### Fitting model

We use an existing model of the direction dependence of the ODMR spectrum of the NV center^14^. The electron spin Hamiltonian of the NV center is given by, $\hat{H} = D \hat{S}_z^2 + E(\hat{S}_x^2-\hat{S}_y^2) + \gamma B\hat{S}_z,$ where $\hat{S}_{x,y,z}$ is the x,y,z component of the spin-1 operator, *D* is the zero-field splitting, *E* is the lattice strain, $\gamma$ is the gyromagnetic ratio of an electron spin, and *B* is the magnetic field strength. At a magnetic field strength of about a few $\mathrm {mT}$, the two resonance frequencies of the NV center can be approximated as $f_{\pm } = D\pm \sqrt{E^2 + (\gamma B)^2}$. The resonance shape of each NV center is approximated as the Lorentzian $L(f_{mw},f_{\pm },\delta \nu _{\pm },C_{\pm }) = C/[(f_{\pm } - f_{mw})^2 + \delta \nu _{\pm }^2]$, where $f_{mw}$ is the applied microwave frequency, $f_{\pm },\delta \nu _{\pm }$, and $C_{\pm }$ are the resonance frequencies, linewidths, and ODMR contrasts, respectively.

We apply the parameters of our experimental setup to the model^14^ to obtain ODMR spectrum $S(f_{mw})$ as,

5 $$\begin{aligned} S(f_{mw}) = \frac{\int _0^{\pi } \kappa (\theta _{NV})P(\theta _{NV}) \int _{0}^{2\pi } [1 - L(f_{mw},f_-,\delta \nu _-,C_-) - L(f_{mw},f_+,\delta \nu _+,C_+)] d\phi _{NV}\sin \theta _{NV}d\theta _{NV}}{2\pi \int _0^{\pi } \kappa (\theta _{NV})P(\theta _{NV})\sin \theta _{NV}d\theta _{NV}} \end{aligned}$$

where $P(\theta _{NV}) \propto \frac{\pi }{12} [32 - \cos \theta _{max}(31 + \cos (2\theta _{max})) - 6\cos (2\theta _{NV})\sin ^2\theta _{max} ]$ is the collection efficiency, and $\kappa (\theta _{NV}) \propto (E_x^2 + E_y^2)\pi (1 + \cos ^2\theta )$ is the light absorption efficiency (more detail is written at S3 in Supplementary information). Equation (5) is used to obtain “model” in Fig. 2c,d.

## Supplementary Information

Supplementary Information.

## Data Availability

The datasets used and analysed during the current study available from the corresponding author on reasonable request.
