# Supplementary material for: Accurate magnetic field imaging using nanodiamond quantum sensors enhanced by machine learning
Source: Sci Rep. 2022 Sep 1;12:13942. doi: 10.1038/s41598-022-18115-w (PMC9436989; doi:10.1038/s41598-022-18115-w)
Supplement: Supplementary file 1 — Supplementary Information. [file 41598_2022_18115_MOESM1_ESM.pdf]

Supplementary information for "Accurate magnetic field imaging using nanodiamond quantum sensors enhanced by machine learning"

Moeta Tsukamoto,[1] Shuji Ito,[1] Kensuke Ogawa,[1] Yuto Ashida,[1,2] Kento Sasaki,[1] and Kensuke Kobayashi[1,2,3]

[1]*Department of Physics, The University of Tokyo, Bunkyo-ku, Tokyo, 113-0033, Japan*

[2]*Institute for Physics of Intelligence, The University of Tokyo, Bunkyo-ku, Tokyo, 113-0033, Japan*

[3]*Trans-scale Quantum Science Institute, The University of Tokyo, Bunkyo-ku, Tokyo, 113-0033, Japan*

Author contact information

Moeta Tsukamoto: moeta.tsukamoto@phys.s.u-tokyo.ac.jp

Kento Sasaki: kento.sasaki@phys.s.u-tokyo.ac.jp

Kensuke Kobayashi: kensuke@phys.s.u-tokyo.ac.jp

S1. STATISTICAL COMPARISON BETWEEN GPR AND PHYSICAL MODEL

We statistically discuss the accuracy comparison between our GPR (machine-learning-based) approach and the physical model. [1].

Figure 2 of the main text shows the difference between the true and the predicted magnetic field analyzed for all the pixels. Figures S1(a)–S1(e) show the histograms of standard deviations obtained for the magnetic field range with every 500 μT. The measurement accuracy increases as the standard deviation becomes smaller and shifts to the left of the graph. GPR has better accuracy than the physical model except for the lowest magnetic field range (0–500 μT) [Fig. S1(a)]. The best accuracy is obtained at 1000–1500 μT [Fig. S1(c)], consistent with Fig. 4(b) in the main text. These results support that GPR is superior for accurate sensing in wide magnetic field ranges.

Why is the physical model more accurate only in the lowest field 0–500 μT shown in Fig. S1(a)? GPR is not suitable since there is only a slight change in this region [Fig. 1(c) in the main text]. On the other hand, the ODMR spectrum is not broad in such a low field, which reduces the uncertainties in the physical model fitting. However, in the higher magnetic field, the directional dependence of the NV center and the frequency dependence of the microwave antenna significantly affect the spectrum, which results in lower accuracy in the model than GPR, as we discuss later.

S2. EFFECTS OF SUBSTRATES ON ACCURACY

We discuss the causes of accuracy suppression when training data obtained from NDE on cover glass is applied to sensing with NDE on silicon. The glass and silicon are different in thermal conductivity, electrical conductivity, and dielectric constant. This fact raises two main effects: the heating by laser and microwave and the frequency characteristics of the microwave antenna.

First, the degree of heating by the laser and microwaves is different between the two materials, resulting in a temperature difference in the NDE on them. It shifts the center of the ODMR spectrum with the coefficient of -74 kHz/K [2].

Second, we explain the impact of the frequency characteristics of the microwave antenna. We use a broadband, large-area microwave antenna [3] to apply a spatially uniform

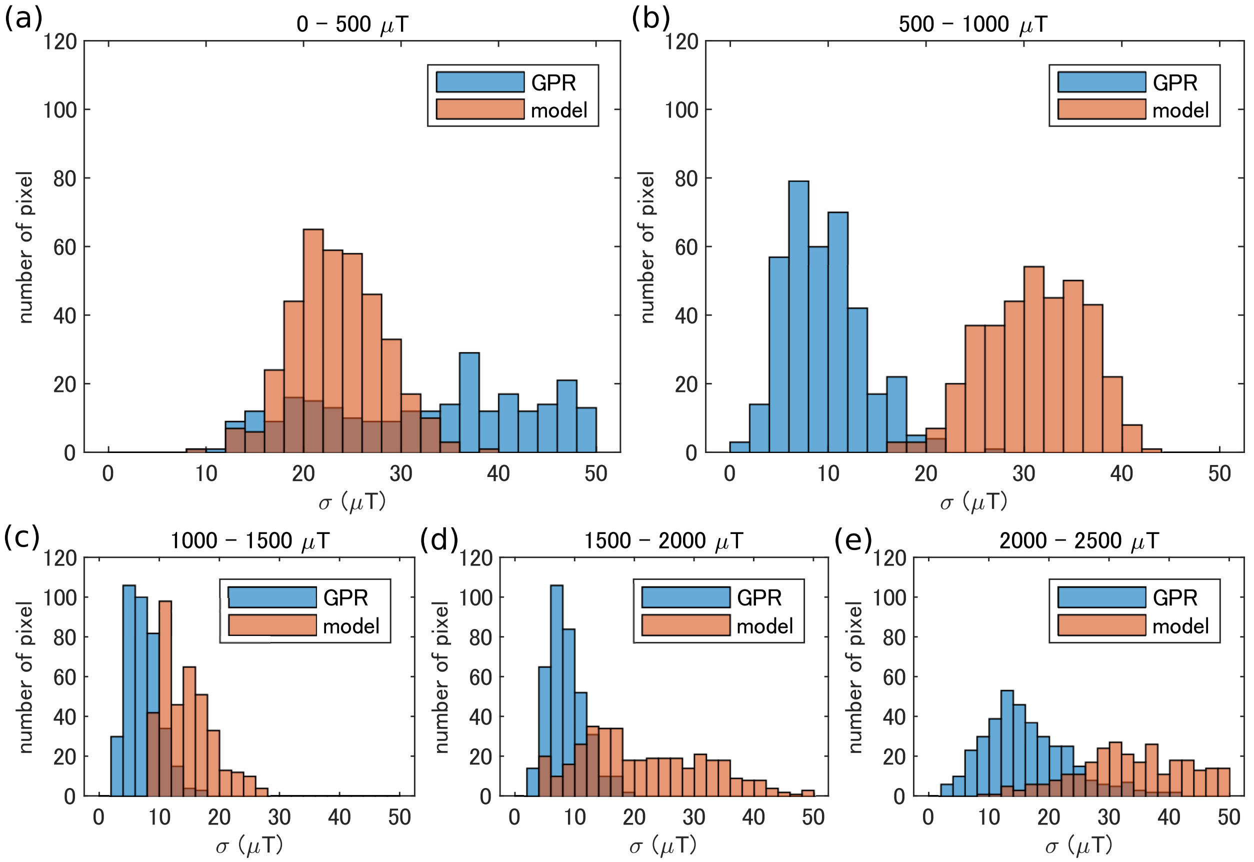

FIG. S1. Standard deviation of the difference between the true and the predicted magnetic field analyzed for all the pixels with GPR and the physical model. The measurement accuracy increases as the histogram shifts to the left of the graph. The magnetic field ranges are (a) 0–500 μT, (b) 500–1000 μT, (c) 1000–1500 μT, (d) 1500–2000 μT, and (e) 2000–2500 μT.

microwave. The frequency characteristics are affected by the conductivity and dielectric constant of the surrounding material. S11 for each measurement condition is shown in Fig. S2. A low S11 indicates less antenna reflection and stronger microwave radiation. The frequency giving the smallest S11 signal corresponds to the resonance frequency. The resonance frequency on the glass is 2.88 GHz, almost the same as the center of the ODMR spectrum. The further away from the center of the ODMR spectrum is, the weaker the microwaves become. This leads to an underestimate of the Zeeman splitting and reduces the accuracy of the physical model. Actually, as shown in Fig. 2(c) in the main text, the physical model gives a lower field value than the true value at high fields (> 1500 μT). On the other hand, the resonance frequency on silicon is 2.97 GHz, which is higher than that on glass. Due to this effect, the ODMR spectrum is shifted to the high-frequency side than it should be.

Since the shift depends on frequency, it also affects the estimation of magnetic field strength.

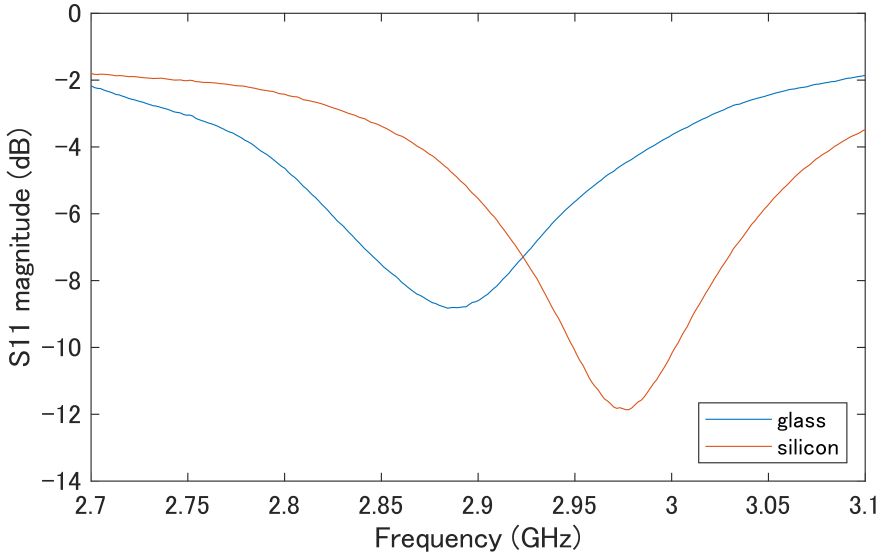

FIG. S2. Measured S11 of our microwave antenna under the two experimental conditions (on glass and on silicon). A Rohde & Schwarz FSH8 spectrum analyzer calibrated with FSH-Z28 is used.

For example, Fig. S3(a) presents the ODMR spectrum obtained in a true magnetic field of 1920 μT. The spectrum of NDE on silicon has a larger contrast on the high-frequency side than that on glass, being consistent with the characteristics of S11. The result on glass shows that the center of the spectrum is on a lower frequency than that on silicon. In the present experiment, black tape is placed underneath the substrate (glass or silicon) for fixing and reducing laser reflection. The different thermal conductivity of glass and silicon contributes differently to the heating, resulting in different spectrum shifts.

We can easily correct the frequency shift caused by these sources. Figure S3(b) depicts the analysis result by incrementally adding a shift to the frequency of the training data. Without any correction (0 MHz), the prediction error is as large as about 150 μT. When the shift of about +6 MHz is incorporated, the prediction error decreases to about 50 μT. The silicon data in Fig. 2(b) in the main text is obtained by applying this appropriate correction.

S3. PHYSICAL MODEL FOR THE ODMR SPECTRUM

We explain the physical model for describing the ODMR spectrum of NDE following Ref. [1]. We consider the resonance frequency, the shape of the resonance, the light absorp-

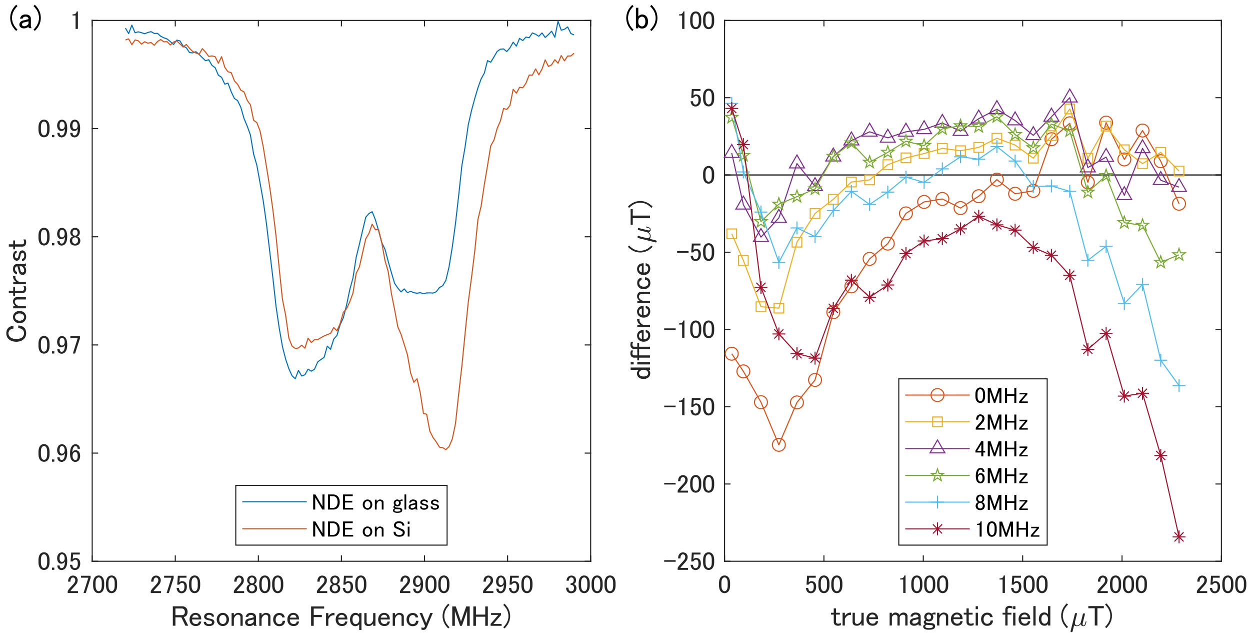

FIG. S3. (a) ODMR spectra of NDE on glass and silicon. (b) Difference between the true magnetic field and the prediction from the test data taken on silicon with training data. The training data is taken on glass. The legends indicate the frequency shift of the training data.

tion efficiency, and the photon collection efficiency.

When the magnetic field is parallel to the NV center's symmetry axis (NV axis), the electron spin Hamiltonian of the NV center is given by,

$$\hat{H} = D\hat{S}_z^2 + E_{\rm s}(\hat{S}_x^2 - \hat{S}_y^2) + \gamma B \hat{S}_z, \tag{S1}$$

where $\hat{S}_{x,y,z}$ are the x,y, and z components of the spin-1 operator, respectively, D is the zero-field splitting, $E_{\rm s}$ is the lattice strain, γ is the gyromagnetic ratio of an electron spin, and B is the magnetic field strength. At a magnetic field strength of a few mT, the two resonance frequencies of the NV center can be approximated as $f_{\pm} = D \pm \sqrt{E_{\rm s}^2 + (\gamma B)^2}$.

The resonance shape of a single NV center is approximated as a Lorentzian $L(f_{\rm mw}, f, \delta\nu, C) = C/[(f - f_{\rm mw})^2 + \delta\nu^2]$, where $f_{\rm mw}$ is the applied microwave frequency, C is the contrast, and $\delta\nu$ is the linewidth.

We carefully define the experimental situation [see Fig. S4(a)] to account for absorption and collection efficiency. Figires S4(b) and (c) define the unit vector parallel to the NV axis $\boldsymbol{e}_{\rm NV}$ with the polar angle $\theta_{\rm NV}$ and the azimuthal angle $\varphi_{\rm NV}$ in the Cartesian coordinate system with the optical axis in the z-axis. For simplicity, we only show the case where the

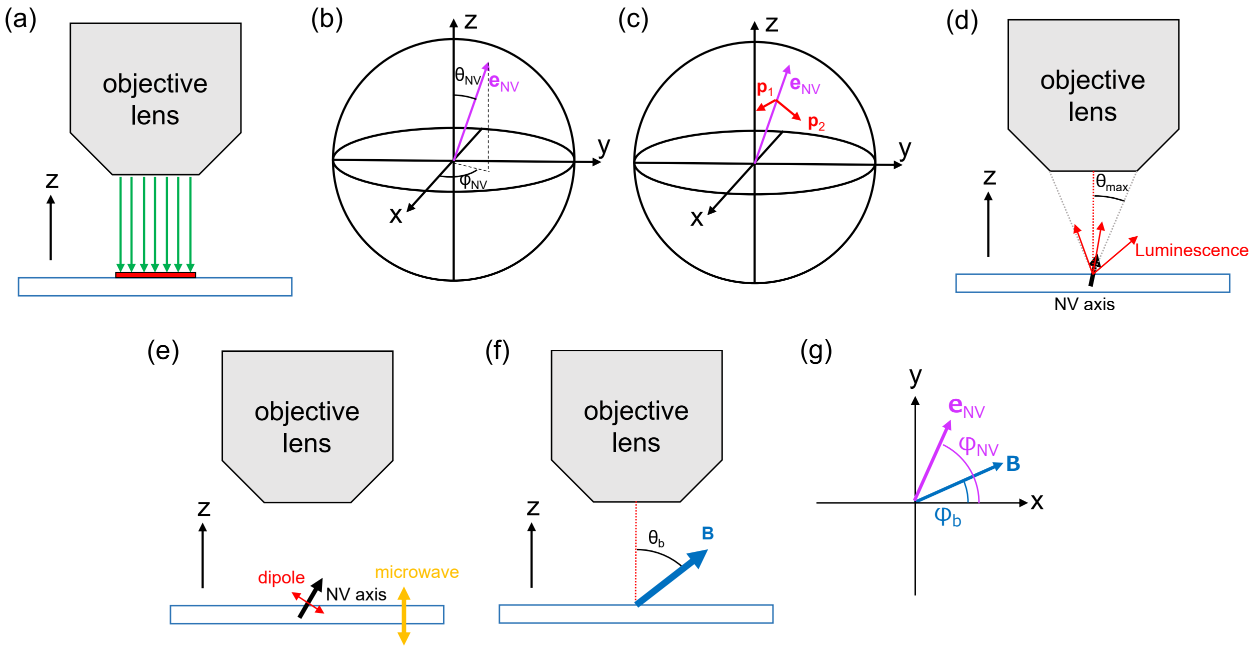

FIG. S4. (a) Schematic of the Köfler type illumination. The excitation light (green arrows) is always irradiated perpendicular to the NDE film (red rectangle) at any position. (b) Unit vector parallel to the NV axis. θ_{NV} and φ_{NV} are defined. (c) NV center's electric dipole moments $\boldsymbol{p}_1$ and $\boldsymbol{p}_2$. (d) Schematic image of luminescence from NV center. The objective lens collects lights within the angle θ_{max}, which is determined by the aperture of the objective lens. (e) Schematic of the microwave field and the NV center's spin dipole. (f) Tilt angle of the magnetic field θ_b with respect to the optical axis. Only the case of $\theta_b = 0$ is considered in this model. (g) Magnetic field direction in the xy-plane when $\theta_b \neq 0$.

magnetic field is applied in the z-axis [Fig. S4(a)], as in previous study [1] and training data measurements.

The light absorption efficiency is proportional to the square of the inner product of the optical transition dipole moment of the NV center and the electric field of the excitation light. The optical transition dipole of the NV center originates from the mixed orbital [4] and has components parallel to the two orthogonal unit vectors $\boldsymbol{p}_1$ and $\boldsymbol{p}_2$. Hereafter, we treat the case as a classical electric dipole moment for simplicity. Despite the C_{3v} symmetry of the NV center, the NV center exhibits an axisymmetric response to linear polarization [5]. Assuming that dipole moments are equivalent in the any perpendicular direction to the NV

axis, we define them as [Fig. S4(c)],

$$\boldsymbol{p}_1 = \frac{\boldsymbol{e}_{\mathrm{NV}} \times \boldsymbol{e}_z}{|\boldsymbol{e}_{\mathrm{NV}} \times \boldsymbol{e}_z|} = \sin\varphi_{\mathrm{NV}}\boldsymbol{e}_x - \cos\varphi_{\mathrm{NV}}\boldsymbol{e}_y, \tag{S2}$$

$$\boldsymbol{p}_2 = \frac{\boldsymbol{e}_{\mathrm{NV}} \times \boldsymbol{p}_1}{|\boldsymbol{e}_{\mathrm{NV}} \times \boldsymbol{p}_1|} = \cos\theta_{\mathrm{NV}}(\sin\varphi_{\mathrm{NV}}\boldsymbol{e}_x + \cos\varphi_{\mathrm{NV}}\boldsymbol{e}_y) - \sin\theta_{\mathrm{NV}}\boldsymbol{e}_z, \tag{S3}$$

where $\boldsymbol{e}_x, \boldsymbol{e}_y$, and $\boldsymbol{e}_z$ are the unit vectors parallel to x-, y-, and z-axis, respectively. The electric field $\boldsymbol{E}$ only in the x and y directions is applied to the NV center with the Köfler type illumination [Fig. S4(a)]. We get the absorption efficiency κ as,

$$\begin{aligned}\kappa(\theta_{\mathrm{NV}}) &\propto \int_0^{2\pi} (|\boldsymbol{p}_1 \cdot \boldsymbol{E}|^2 + |\boldsymbol{p}_2 \cdot \boldsymbol{E}|^2) d\varphi_{\mathrm{NV}} \\ &= (E_x^2 + E_y^2)\pi(1 + \cos^2\theta_{\mathrm{NV}}),\end{aligned} \tag{S4}$$

where $\boldsymbol{E} = E_x\boldsymbol{e}_x + E_y\boldsymbol{e}_y$. The absorption efficiency is higher when the NV axis is parallel to the optical axis. This means that photons coming from the direction of the NV axis are absorbed better.

The photon collection efficiency is also determined by the electric dipole moment [6]. The energy of light emitted from a point dipole $\boldsymbol{p}$ to a position $\boldsymbol{r}$ well away from its wavelength is,

$$|\boldsymbol{S}| \propto |\boldsymbol{e}_r \times \boldsymbol{p}|^2 / r^2, \tag{S5}$$

where $\boldsymbol{r} = r\boldsymbol{e}_r = r(\cos\phi_r \sin\theta_r \boldsymbol{e}_x + \sin\phi_r \sin\theta_r \boldsymbol{e}_y + \cos\theta_r \boldsymbol{e}_z)$. The light is more likely to be emitted perpendicular to $\boldsymbol{p}$, i.e., in the direction of the NV axis. The measurable angle of an objective lens is determined by NA$= \sin\theta_{\mathrm{max}}$ [Fig. S4(d)]. The light collection efficiency is obtained as,

$$P(\boldsymbol{p}) \propto \int_0^{\theta_{\mathrm{max}}} \int_0^{2\pi} |\boldsymbol{S}| r^2 \sin\theta_r d\phi_r d\theta_r. \tag{S6}$$

By replacing the dipole direction with the tilt of the NV center, we can integrate Eq. (S6) as,

$$P(\theta_{\mathrm{NV}}) \propto \frac{\pi}{12}[32 - \{31 + \cos(2\theta_{\mathrm{max}})\}\cos\theta_{\mathrm{max}} - 6\cos(2\theta_{\mathrm{NV}})\sin^2\theta_{\mathrm{max}}]. \tag{S7}$$

Putting all the components together, we obtain the result for ODMR spectrum $S(f_{\mathrm{mw}})$ as,

$$S(f_{\mathrm{mw}}) = \frac{\int_0^{\pi} \kappa(\theta_{\mathrm{NV}}) P(\theta_{\mathrm{NV}}) \int_0^{2\pi} [1 - L(f_{\mathrm{mw}}, f_-, \delta\nu_-, C_-) - L(f_{\mathrm{mw}}, f_+, \delta\nu_+, C_+)] d\varphi_{\mathrm{NV}} \sin\theta_{\mathrm{NV}} d\theta_{\mathrm{NV}}}{2\pi \int_0^{\pi} \kappa(\theta_{\mathrm{NV}}) P(\theta_{\mathrm{NV}}) \sin\theta_{\mathrm{NV}} d\theta_{\mathrm{NV}}} \tag{S8}$$

where $f_{\pm}, \delta\nu_{\pm}$, and $C_{\pm}$ are the resonance frequencies, linewidths, and ODMR contrasts of the two resonances ($\pm$), respectively. Equation (S8) agrees with the previous model [1]. Equation (S8) is used for fitting in Figs. 2(c) and 2(d) of the main text.

Figures S5 (a) and (b) show two examples of the fitting to the physical model. While the estimated magnetic field deviates from the true value, the model seems to reproduce the experimental spectrum at a low field reasonably ($B = 547.1 \pm 0.12$ μT) [Fig. S5(a)]. However, this is not the case at a high field ($B = 2103 \pm 0.12$ μT) [Fig. S5(b)]. This difference indicates that the complexity of the experimental conditions is not fully reflected in the model. Specifically, we describe two contributions as follows. First, as mentioned in the previous section, the frequency dependence of the microwave strength radiated from a resonator-type microwave antenna exists. Since the resonance spectrum of an NDE is continuous over a wide range, even a small frequency dependence of the antenna distorts the spectral shape. Second, microwave absorption is dependent on the directions [Fig. S4(e)]. It is easy to absorb the microwave field of the component perpendicular to the NV axis. The contrast of the NV center, which is oriented in the same direction as the microwave field, is reduced, resulting in inaccurate magnetic field estimation.

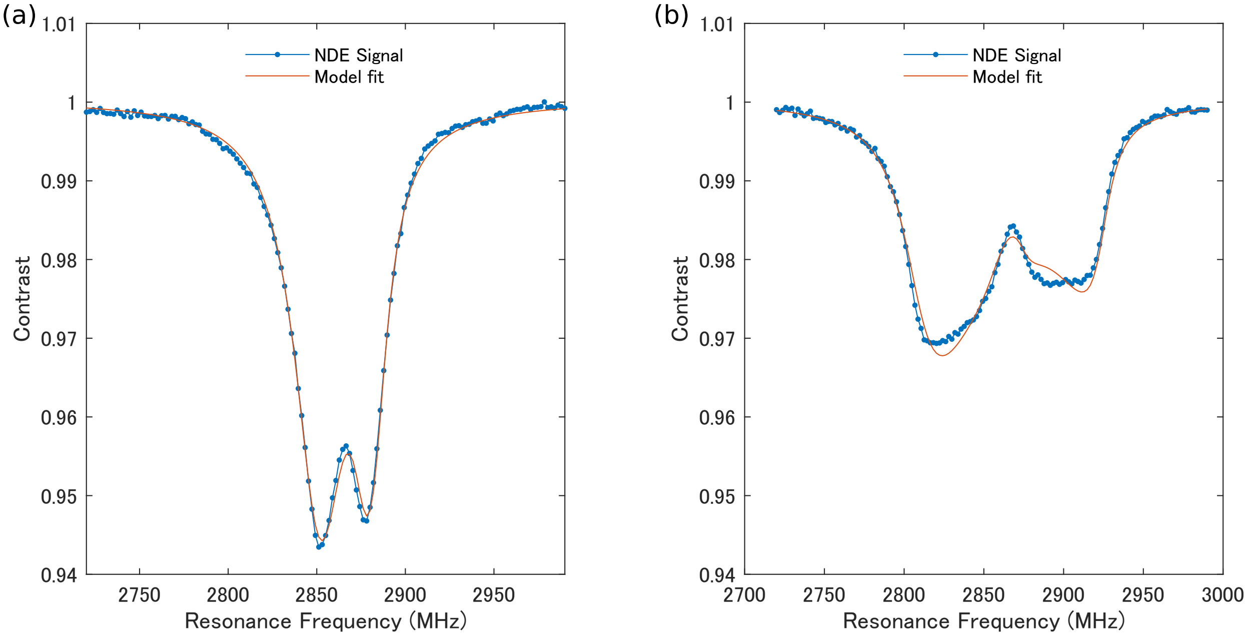

FIG. S5. Examples of fitting with the physical model [Eq. (S8)]. (a) The ODMR spectrum at true magnetic field $B = 547.1 \pm 0.12$ μT. The estimated value is 582.2 ± 4.6 μT. (b)The ODMR spectrum at true magnetic field $B = 2103 \pm 0.12$ μT. The estimated value is 2062 ± 8 μT.

In this physical model, the magnetic field direction is assumed to be in the optical (z-axis) direction. If the magnetic field direction deviates from the z-axis [Fig. S4(f)], the dependence on the azimuth angle needs to be considered [Fig. S4(g)]. The light absorption efficiency and photon collection efficiency must be integrated against the azimuth angle at the same time as the resonance shape, making the analytical calculation more difficult. The actual effects that can occur are discussed in the next section.

S4. DIRECTIONAL DEPENDENCE OF PREDICTED MAGNETIC FIELD

We observe the three-dimensional field direction dependence of ODMR spectra [see Fig. 2(b) in the main text]. There are three causes of the GPR prediction difference in the z- and xy- components and an additional one cause of the difference in the x- and y-directions. We explain these four causes in order below.

The first is the absorption efficiency of the NV center. Due to Köfler type illumination, the NV symmetry axis in the z-direction ($\theta_{NV} = 0$) is easily excited.

The second is the light collection efficiency [6]. The objective lens has a better light collection efficiency in the z-direction, and the NV has a high photon emission probability in the direction of the NV symmetry axis.

The third is the angular dependence of the microwave absorption efficiency, as shown in Fig. S4(e).The microwave field perpendicular to the NV symmetry axis drives the NV center's magnetic resonance [5]. In our experiment, we apply linearly polarized microwaves in the z-direction, so it is easy to obtain ODMR contrast of the NV center in the x and y directions. This is the opposite contribution to the first and second causes.

The fourth is the difference in the absorption efficiency due to the linearly polarized component of the excitation light. The NV center is more easily excited when its symmetry axis is perpendicular to linear polarization than in the parallel case. Since the excitation light used in this experiment is elliptically polarized, the linearly polarized component of the excitation light causes a bias in the estimated magnetic field in the x- and y-directions.

All these effects contribute to the results. In Fig. 2(b) in the main text, GPR predicts lower values than the true magnetic field when the magnetic field is applied in the x- and y-directions. The contribution of the third and fourth causes is estimated to be significant under our experimental conditions. We also confirmed that the difference for the x- and

y-directions is modulated by the light polarization (data not shown).

S5. ADDITIONAL DATA FOR MAGNETIC FIELD IMAGING

We adopt Ampere's law for fitting the magnetic field distribution in Fig. 3(b). The only fitting parameters are the positions of the copper wire in the x- and z-directions. In addition to the data shown in Fig. 3(a) of the main text, we measure magnetic field distributions at three different current values. Figures S6(a), S6(b), and S6(c) show the obtained magnetic field distributions. At all current values, the magnetic fields are larger at the position closer to the copper wire. The averaged results in the y-direction are summarized in Fig. S6(e). All the experimental results are consistent with Fig. 3(b) in the main text; We can perfectly reproduce them using the same wire position.

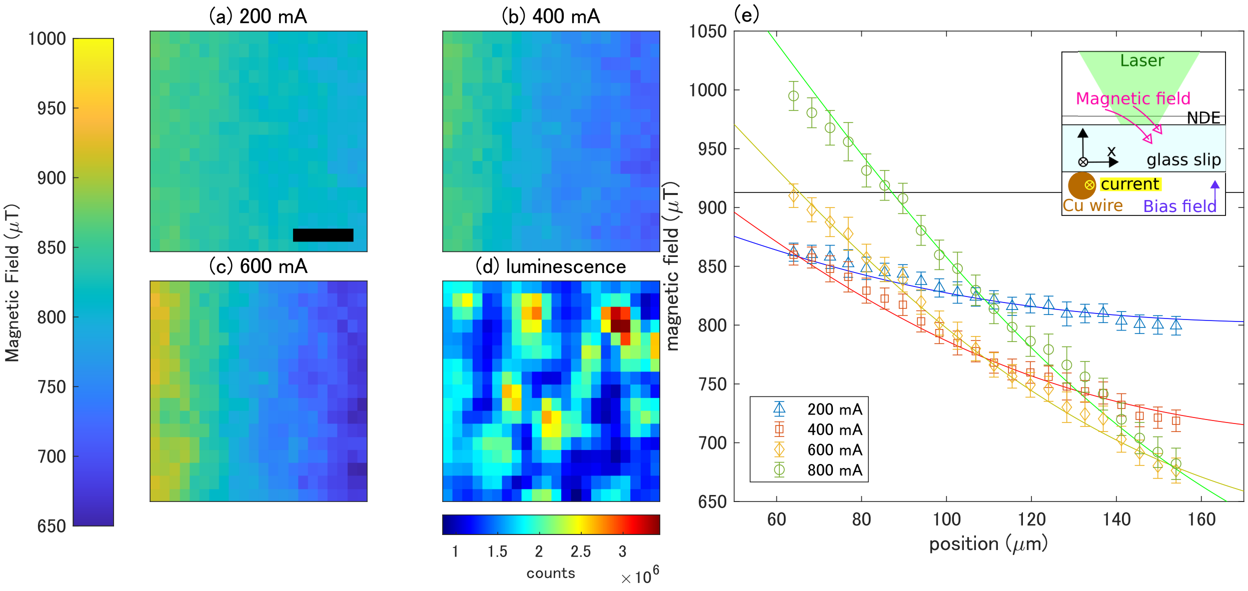

FIG. S6. Magnetic field distributions at currents of (a) 200 mA, (b) 400 mA, and (c) 600 mA. The scale bar in (a) is 20 μm. (d) Photoluminescence intensity distribution. (e) Magnetic field distribution averaged in the y-direction for 200 mA, 400 mA, 600 mA, and 800 mA. The data for 800 mA is the same as that shown in Fig. 3(b) in the main text. The colored solid lines are the results of the fitting based on Ampere's law. The horizontal black solid line at 912.8 μT indicates the bias field strength. (inset) Measurement configuration, where the bias field applied in the z-direction and the field generated by the current through the wire are simultaneously felt by the NDE.

Figure S6(e) tells that the data of different current values cross each other. This is because the direction of our bias magnetic field and the magnetic field generated by the current are opposite, so they partially cancel each other.

If we look carefully at the magnetic field distribution, we identify that there are spatial fluctuations. It is due to the speckle of the excitation light passing through the multimode fiber. Figure S6(d) shows the photoluminescence intensity distribution of the NV center. The area with low intensity is the area with low excitation light intensity. The signal-to-noise ratio in such regions is low, partially degrading the magnetic field accuracy and sensitivity, which appears as the outlier in fig. 4(c) in the main text. Note that we have confirmed that such inhomogeneity does not significantly impact the machine-learning outcome.

[1] C. Foy, L. Zhang, M. E. Trusheim, K. R. Bagnall, M. Walsh, E. N. Wang, and D. R. Englund, Wide-field magnetic field and temperature imaging using nanoscale quantum sensors, ACS Applied Materials & Interfaces **12**, 26525 (2020).

[2] V. M. Acosta, E. Bauch, M. P. Ledbetter, A. Waxman, L.-S. Bouchard, and D. Budker, Temperature dependence of the nitrogen-vacancy magnetic resonance in diamond, Physical Review Letters **104**, 070801 (2010).

[3] K. Sasaki, Y. Monnai, S. Saijo, R. Fujita, H. Watanabe, J. Ishi-Hayase, K. M. Itoh, and E. Abe, Broadband, large-area microwave antenna for optically detected magnetic resonance of nitrogen-vacancy centers in diamond, Review of Scientific Instruments **87**, 053904 (2016).

[4] J. R. Maze, A. Gali, E. Togan, Y. Chu, A. Trifonov, E. Kaxiras, and M. D. Lukin, Properties of nitrogen-vacancy centers in diamond: the group theoretic approach, New Journal of Physics **13**, 025025 (2011).

[5] T. P. M. Alegre, A. C. Torrezan, and G. Medeiros-Ribeiro, Microstrip resonator for microwaves with controllable polarization, Applied Physics Letters **91**, 204103 (2007).

[6] V. R. Horowitz, B. J. Aleman, D. J. Christle, A. N. Cleland, and D. D. Awschalom, Electron spin resonance of nitrogen-vacancy centers in optically trapped nanodiamonds, Proceedings of the National Academy of Sciences **109**, 13493 (2012).
